# Examining Changes in Active Coping Strategies Among Latinx Adolescents: Latent Growth Curve

**DOI:** 10.1002/jad.70052

**Published:** 2025-09-15

**Authors:** Rayni Thomas, Rajni L. Nair, Melissa Y. Delgado

**Affiliations:** ^1^ The Nebraska Center for Research on Children, Youth, Families and Schools, Nebraska Academy for Methodology, Analytics & Psychometrics University of Nebraska‐Lincoln Lincoln Nebraska USA; ^2^ College of Integrative Sciences and Arts Arizona State University Phoenix Arizona USA; ^3^ College of Agriculture & Life Sciences, Norton School of Family and Consumer Sciences The University of Arizona Tucson Arizona USA

## Abstract

**Introduction:**

As active coping strategies support the development and adjustment of Latinx adolescents, it is important to identify periods of growth and decline. Yet, no work has examined trajectories of active coping within the unique developmental context of Latinx adolescents. The purpose of this study was to (a) examine changes in active coping strategies during the transition from middle to high school (Goal 1) and (b) explore variations in the trajectories of active coping by gender and nativity (Goal 2) among Latinx adolescents.

**Method:**

Using latent growth curve analysis, trajectories of active coping strategies across the transition from middle to high school, (i.e., 3 time points, 8th grade, 9th grade, and 10th grade) were examined among 288 Latinx adolescents at ages 13 to15 at Time 1 (*M*_*ageT1*_ = 13.69, SD_*ageT1*_ = 0.56).

**Results:**

The study found stability in the trajectory of active coping during the transition from 8th to 9th grade, but a significant curvilinear decline in active coping during the transition from 9th grade to 10th grade; there were no differences in the trajectory of active coping by gender and nativity.

**Conclusion:**

The findings suggest that the transition from 9th grade to 10th grade is a sensitive period that clinical and educational program administrators may want to target when focusing on developing active coping among Latinx adolescents.

Active coping strategies, or ways in which individuals engage cognitive or behavioral efforts to understand and resolve problems (Ayers et al. 1996), are an important asset that supports the development and adjustment of Latinx adolescents (e.g., Brietzke and Perreira 2017). Although several types of coping strategies (e.g., emotion‐focused coping) are used to manage feelings of distress (Gonzales et al. 2001), active coping strategies are more frequently employed during adolescence (Seiffge‐Krenke and Beyers 2005). Importantly, active coping is not a static construct; rather it is recalibrated and adjusted as youth mature and navigate their changing ecologies (Lerner et al. 2012). The life course perspective postulates developmental turning points, moments of heightened change in individual‐level processes, provoke growth or decline of assets (Elder 1998), such as active coping. The transition from middle to high school may represent a developmental turning point (Sawyer et al. 2018; Constante et al. 2021). Understanding the trajectory of active coping during this key transition is salient to identify when to provide adequate and appropriate resources that foster problem‐solving skills facilitating active coping among Latinx adolescents. However, no work has examined the trajectory of active coping within the unique developmental contexts of Latinx adolescents.

Examining the trajectory of active coping within the unique developmental contexts of Latinx adolescents provide an avenue to better understand within‐group variations. Prior work suggests there are nuanced cultural variations between foreign‐born and U.S.‐born Latinx populations (Buckingham and Suarez‐Pedraza 2019), which may inform differences in the developmental trajectory of active coping. Also, literature focused on biological differences between boys and girls (i.e., Blakemore and Choudhury 2006) provides evidence for variations in the developmental trajectory of active coping. As such, the developmental trajectories of active coping may differ by nativity and gender. The purpose of this study was to (a) examine changes in active coping strategies during the transition from middle to high school (Goal 1; see Figure 1) and (b) explore variations in the trajectories of active coping by gender and nativity (Goal 2; see Figure 2) among Latinx adolescents.

**Figure 1:**
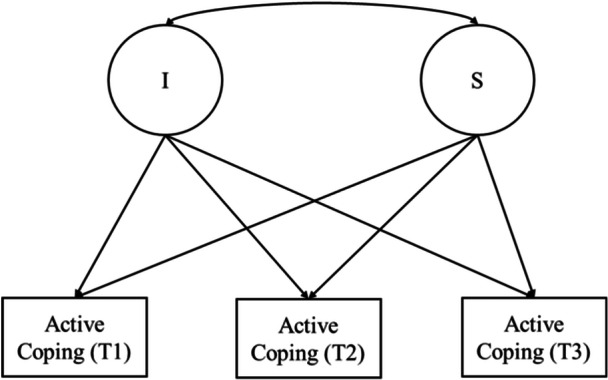
Conceptual model of the trajectory of active coping strategies (Goal 1). *Note:* I = Intercept; S = Slope.

**Figure 2:**
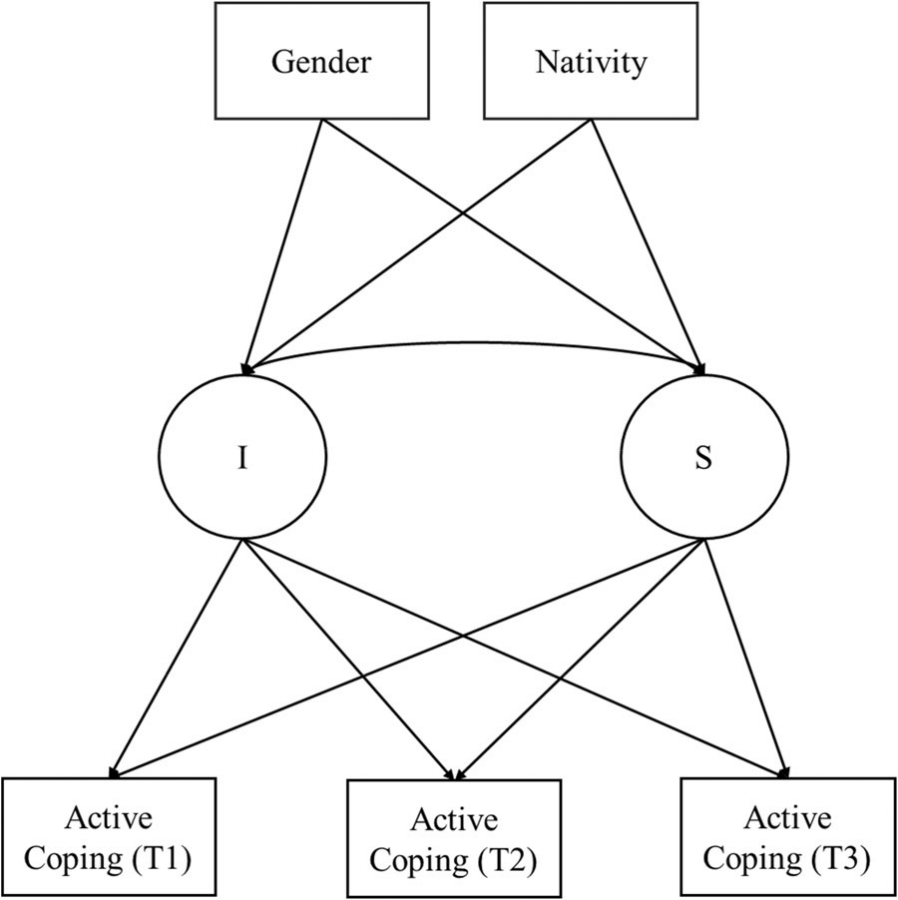
Conceptual model of the difference in the trajectory of active coping strategies by gender and nativity (Goal 2). *Note:* I = Intercept; S = Slope.

## 1 Theoretical Background

The life course perspective suggests turning points occur when developmental, social, and environmental changes provide either an abundance or a lack of opportunities, invoking growth or decline in assets (Alexander et al. 1988; Elder 1998). As adolescents move from middle to high school they may have limited access to established social support systems (e.g., peer groups, teachers, school administrators) or greater access to school resources (e.g., counseling services; Cohen et al. 2017; Dupéré et al. 2015). For example, among Latinx adolescent populations the transition from middle to high school may provide greater access to extended family (e.g., older cousins) networks facilitating an increase in social support systems that draw on collectivistic cultural ideologies for problem solving when encountering challenges in the school context. Additionally, increased cognitive capacities such as metacognition, the ability to plan ahead, and the ability to think about future consequences of an action, are further scaffolded through course work and projects in high school (Lazzara 2020), which may inform the trajectory of active coping strategies. For example, the ability to plan ahead allows adolescents to identify possible problems and think of solutions ahead of time, circumventing consequences of not finding a resolution to everyday challenges. Regarding gender, due to hormonal differences, brain development (i.e., amount of gray vs. white matter) tends to take longer for middle school boys than girls (Blakemore and Choudhury 2006). Indeed, middle school boys had more gray matter than girls, as boys aged, their white matter increased, and girls' white matter remained relatively stable (Blakemore and Choudhury 2006). As white matter is indicative of neural connections and better cognitive capacities (Blakemore and Choudhury 2006), differences in brain development between boys and girls may relate to variations in the trajectory of active coping strategies by gender. Whereas sociocultural norms (e.g., endorsing individualistic vs. collectivistic values; Kayser 2012) between U.S. and Latinx cultures may result in differences in the trajectory of active coping by nativity.

## 2 Trajectories of Active Coping

Past works, among multi‐ethnic U.S. adolescent samples, examining changes in the trajectory of active coping showed mixed findings. Sandler et al. (1994) observed increases in active coping across two time points (i.e., 5.50 months between T1 and T2) among predominantly White (i.e., 86% White) early adolescents (average age 10). The changes in active coping found by Sandler et al. (1994) may reflect the rapid cognitive development that characterizes early adolescence and enables youth to more effectively engage in problem‐focused strategies. Indeed, constructs similar to active coping (i.e., primary control engagement coping) that focus on problem‐solving have also been found to increase during adolescence in extant literature. Evans et al. (2015) found a consistent increase in primary control engagement coping which is defined as “attempts to directly change the situation or one's emotional reaction to the situation and includes problem‐solving, emotional expression, and emotion regulation” (p. 2). Specifically, the study found primary control engagement coping strategies (i.e., composite of active, planning, and seeking social support coping) consistently increased across four time points spanning early to late adolescence among majority White (i.e., 69.6% White, 21.6% African American) adolescents (i.e., average age 12; Evans et al. 2015).

In contrast, other work found that active coping strategies decreased from ages 14 to 17, then increased steadily until about age 23, after which point active coping decreased slightly among a multi‐ethnic (i.e., at T1 58% White, 11% Latinx) sample (Vannucci et al. 2018). The mixed findings in prior literature highlight a need to further extend research in ways that allow for a nuanced understanding of the trajectory of active coping strategies within differing developmental contexts (e.g., within cultures that endorse collectivistic values and beliefs or among ethnic‐racial minoritized adolescents navigating experiences of ethnic‐racial discrimination). Indeed, the reviewed extant literature examining the development of active coping utilized predominantly White adolescent samples. However, the developmental contexts of White youth do not fully reflect the same experiences youth of color may encounter within their developmental contexts (Coll et al. 1996). As such there is a need to understand variations in the trajectory of active coping within the developmental contexts of youth of color by utilizing ethnically homogeneous samples.

In fact, empirical work suggests that Latinx youths' experiences with unique sociocultural stressors (e.g., ethnic‐racial discrimination) increases during adolescence, generally, (Constante et al. 2021) and in high school specifically (Benner and Graham 2011). In this way, Latinx adolescents are not only experiencing universal stressors (e.g., increased course work), common to all adolescents, but unique sociocultural stressors during the transition from middle to high school. Concomitantly, Latinx adolescents may utilize collectivistic cultural strengths such as seeking advice from elders, or close extended family connections to problem solve and navigate stressors (Brietzke and Perreira 2017). Such experiences form unique developmental contexts, yet there is no work examining changes in active coping during the transition from middle to high school specifically among Latinx adolescents. The current study aims to extend prior work to better understand active coping trajectories by estimating normative changes in active coping during the transition from middle (i.e., 13 years old) to high school (i.e., 16 years old) among Latinx adolescents.

## 3 Variation by Gender and Nativity

Prior work suggests that there are developmental differences by gender. Utilizing a predominately White adolescent (i.e., 65%) sample, past work found that although both girls' and boys' active coping declined over time, the decline was significantly steeper for boys' (Flannery et al. 2018). One potential explanation for this difference in trajectories is that boys lag behind girls in some aspects of executive functioning (Flannery et al. 2018), starting in the middle school years (Lazzara 2020). As executive functioning is the brain's ability to think about the future, plan, and include other cognitive skills needed to engage in problem solving activities central to implementing active coping strategies, developmental delays in executive functioning among boys may translate to gender differences in the trajectory of active coping. As such, to extend existing literature, the current study explored whether there were differences in the trajectory of active coping by gender as Latinx girls and boys transition to high school.

Additionally, there may be differences in active coping by nativity. Past work has found, while living in the United States, foreign‐born Latinx adolescents may adapt or acculturate to American culture and become more individualistic (Buckingham and Suarez‐Pedraza 2019). Indeed, prior work among Latinx middle school students found that the use of active coping strategies increased as youths' level of acculturation increased (Gudiño et al. 2018). As such, active coping may differ over time by nativity as foreign‐born Latinx adolescents' levels of acculturation may change over time. Yet, no studies have examined differences in active coping trajectories between foreign‐born and U.S.‐born Latinx adolescents. Thus, the current study will extend existing literature by examining variations of active coping trajectories by nativity among Latinx adolescents.

## 4 Method

### 4.1 Procedure

The data for the current study comes from a 3‐year longitudinal study among Latinx middle school students living in the U.S. Southwest. To participate in the study, families needed to have: (a) a child in the eighth grade, and (b) biological mothers and/or biological or long‐term (i.e., before age 2) adoptive fathers living in the students' homes and had origins in Latin American countries. All survey answers were recorded using the online data collection site Qualtrics. Participating families received a $25 gift‐card to a local store for their time spent completing the surveys. All study procedures were reviewed and approved by the University's Institutional Review Board.

### 4.2 Participants

The current study includes 288 adolescents. Participants' ages at Time 1 ranged from 13 to 15 years old (*M* = 13.69, *SD* = 0.56); 5 participants did not report their age. The sample consists of more girls (48%) than boys (41%), some participants did not report their gender (11%). Participants' reported birthplaces included the U.S. (86%), Mexico (9%), and Latin American countries (5%); one participant was not sure of their birthplace (0.30%). Participants' reported ethnicity included Hispanic (45%), Mexican (20%), Mexican American (19%), Latinx (7%), White (3%), White/Hispanic (2%), Chicanx (2%), African American/Mexican (0.60%), Mixed/Other (0.60%), Native American (0.60%), African American/Latinx (1%), Other Hispanic (0.30%), and African American (0.30%); one participant did not report their ethnicity.

Mothers of adolescents in the current study reported on various indicators of socioeconomic status. Specifically, 270 mothers provided information on their education level and participation in government assistance programs. Eighteen mothers did not complete a parent survey. Among the mothers that completed a survey, 40% reported less than a high school diploma, 15% reported a GED or high school diploma, 39% reported vocational school/some college or Associates/Bachelor's degree, and 6% reported Master's degree or Doctorate. Also, utilizing a select all that apply format, mothers were asked to report on the government assistance programs they received. Approximately 19% of mothers reported receiving Food Stamps, 34% reported receiving Medicaid, 6% reported receiving Earned Income Tax Credit, 0.3% reported receiving TANF, and 1.4% reported receiving HeadStart/Early HeadStart services.

### 4.3 Measures

#### 4.3.1 Active Coping Strategies

The active coping subscale from the Children's Coping Strategies Checklist (Ayers et al. 1996; Sandler et al. 1997) was used and consisted of 12 items (e.g., “You did something to solve the problem.”). The response options for the scale were from 1 = *almost never or never* to 5 = *almost always or always*. This measure has demonstrated adequate reliability and validity in Mexican‐origin adolescent samples (Liu et al. 2011). Also, the active coping strategies scale was tested for longitudinal invariance across times one through three, and group invariance at time one by gender and nativity. As such, configural (equal form), metric (equal factor loadings), and scalar (equal item intercepts) models were compared for good fit and CFI change (ΔCFI < 0.01) before longitudinal analysis (Brown 2006). Time invariance of the active coping strategies scale at T1–T3 was established at the configural, metric, and scalar levels. Group invariance of the active coping strategies scale at T1 by gender and nativity was established at the configural, metric, and scalar levels. At T1 the Cronbach's alpha was 0.93, at T2 it was 0.90, and at T3 it was 0.92. Scale scores were created by calculating the average score across items.

#### 4.3.2 Background Characteristics

Participants self‐reported gender (i.e., “What is your gender?”) and nativity (i.e., “Where were you born?”). Dummy coded: 0 = *boys*, 1 = *girls*; 0 = *foreign‐born adolescents*, 1 = *U.S.‐born adolescents*.

### 4.4 Analytic Plan

IBM SPSS Statistics version 28 (IBM Corp 2021) was used for descriptive statistics and correlations. To address the study aims, a series of latent growth curve models (LGCM) were examined using Mplus version 8.3 (Muthén and Muthén 1998–2019) and the full information maximum likelihood estimator (FIML; Enders 2010) was used to account for missing data. Three time points were used to examine changes in active coping strategies during the transition from middle to high school. Model fit was evaluated using multiple fit indices, including the chi‐square (*χ* ^*2*^) value, the Comparative Fit Index (*CFI* > 0.90), the Root Mean Square Error of Approximation (*RMSEA* < 0.10), and the Standardized Root Mean Square Residual (*SRMR* < 0.10; Hu and Bentler 1999). To address Goal 1, first a random‐intercept or no growth model was estimated to determine whether active coping changed over time. Second, a linear growth curve model was examined by fixing the paths of the latent linear slope term to 0, 1, and 2. The next model estimated was a quadratic model with the quadratic variance constrained to zero. The quadratic mean shows the trajectory of the linear slope with each additional unit of time. The best‐fitting LGCM model was determined by evaluating the model fit indices, and the change in *CFI* (a change greater than 0.01 establishes a significant difference between models). For Goal 2, variations in active coping trajectories by adolescent gender and nativity were examined by including gender and nativity as independent variables predicting the intercept and slope of the best‐fitting LGCM.

## 5 Results

Bivariate correlations and descriptive statistics for all study variables are presented in Table 1.

**Table 1:** Correlations, means (M), and standard deviation (SD) for all study variables (*N* = 288).

Variables	1.	2.	3.	4.	5.
1. Active Coping (T1)	—				
2. Active Coping (T2)	0.43***	—			
3. Active Coping (T3)	0.27***	0.35***	—		
4. Gender	−0.01	0.05	−0.04	—	
5. Nativity	0.05	−0.04	0.10	−0.05	—
*M*	3.98	3.97	3.67	0.54	0.87
*SD*	0.76	0.64	0.70	0.50	0.34
*Skewness*	−0.78	−0.79	−0.31	−0.15	−2.16
*Kurtosis*	0.38	1.06	0.29	−1.99	2.67

*Note*: *M* = Mean; *SD* = Standard Deviation; Adolescent gender coded 1 = girls, 0 = boys; Adolescent nativity coded 1 = U.S.‐born, 0 = foreign‐born.

* *p* < 0.05

** *p* < 0.01

*** *p* < 0.001.

### 5.1 Goal 1: Trajectories of Active Coping

**Figure 3:**
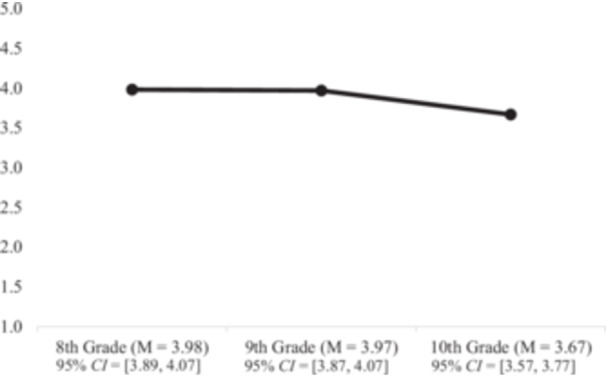
Quadratic Change in Active Coping Strategies From 8th to 10th Grade. *Note*: Utilizing simple means, this figure visually represents the significant quadratic change in active coping strategies; *M* = mean; *CI* = Confidence Interval.

The random intercept (*χ* ^*2*^ (6) = 44.64, *p* = 0.00; *CFI* = 0.25; *RMSEA* = 0.15; *SRMR* = 0.30), and linear growth (*χ* ^*2*^ (3) = 10.26, *p* = 0.02; *CFI* = 0.86; *RMSEA* = 0.09; *SRMR* = 0.17) models showed poor fit. The quadratic growth model met standards for acceptable fit (*χ* ^*2*^ (2) = 1.06, *p* = 0.59; *CFI* = 1.00; *RMSEA* = 0.00; *SRMR* = 0.09). The change in *CFI* between the random intercept and quadratic growth models (*∆CFI* = 0.75), as well as between the linear and quadratic growth models (*∆CFI* = 0.14) indicated a significant difference between models. As the quadratic model met standards for good fit and is significantly different from the random intercept and linear model, the quadratic model was retained as the final model for this study. Parameter estimates for the quadratic growth model are presented in Table 2. Results showed the linear mean was not significant, but the linear variance was significant, indicating there was no significant change in active coping between T1 and T2 for the overall sample but there was individual variation (i.e., differences in the trajectory of active coping for each person) in the linear slope between T1 and T2. The quadratic mean was significant and negative, suggesting that between T2 and T3 the linear slope showed a significant curvilinear downward trajectory (see Figure 3).

**Table 2:** Parameter estimates for the quadratic latent growth model of active coping.

Growth Curve Component	*B (SE)*
Intercept mean	3.98 (0.04)***
Intercept variance	0.31 (0.05)***
Linear mean	0.14 (0.10)
Linear variance	0.07 (0.03)**
Quadratic mean	−0.15 (0.05)**
Quadratic variance^a^	0.00 (0.00)

*Note*: Estimates are presented as *B(SE)*.

^a^ variance was set to 0.

** *p* < 0.01

*** *p* < 0.001.

### 5.2 Goal 2: Gender and Nativity Variation

Differences in the quadratic growth model by gender and nativity were tested. Both the gender (*χ* ^*2*^ (3) = 4.39, *p* = 0.22; *CFI* = 0.97; *RMSEA* = 0.04; *SRMR* = 0.08), and nativity (*χ* ^*2*^ (3) = 3.87, *p* = 0.28; *CFI* = 0.98; *RMSEA* = 0.03; *SRMR* = 0.08) quadratic growth models met standards for model fit. However, there were no significant differences found by gender or nativity in the quadratic growth model, such that there was no significant intercept, *b* = 0.01, *SE* = 0.01, *p* = 0.49, or linear slope differences, *b* = −0.00, *SE* = 0.01, *p* = 0.58, by gender. Nor was there any significant intercept, *b* = 0.07, *SE* = 0.12, *p* = 0.56, or linear slope difference, *b* = 0.01, *SE* = 0.08, *p* = 0.93, by nativity. Differences by gender and nativity were not examined for the quadratic trajectory as the quadratic variance was constrained to zero.

## 6 Discussion

No work has examined the trajectory of active coping strategies within the unique developmental contexts of Latinx adolescents as they transition into high school. The current study extended existing works by examining (a) the trajectory of active coping strategies during the transition from middle to high school and (b) whether the trajectory differed by gender and nativity among Latinx adolescents. This study expands the fields' understanding of the trajectory of active coping within the unique developmental contexts of Latinx adolescents.

### 6.1 Goal 1: Trajectories of Active Coping

The findings of the current study align with prior work that shows a general decline in active coping during the high school years (Flannery et al. 2018; Vannucci et al. 2018). One plausible explanation for why the downward shift occurred for youth across 9th to 10th grade instead of 8th to 9th is rooted in cognitive and social development. Due to the development of cognitive functioning starting in the middle school years (Lazzara 2020), Latinx adolescents are able to think in more complex ways about their futures during the transition from 9th to 10th grade compared to the transition from 8th to 9th grade. As Latinx adolescents transition from 9th to 10th grade, and grow closer to adulthood, their values and perspectives may change as they learn and think about more global concepts (e.g., justice, history, and politics; Sawyer et al. 2018). Latinx adolescents increased social perspective taking, awareness and future oriented thinking in 9th to 10th grade may encourage them to face realities of being marginalized students in a world of White supremacy. Such changes between the 9th and 10th grade may require Latinx adolescents to rely more on other types of coping strategies (e.g., emotion‐focused coping), rather than active coping.

Additionally, as Latinx adolescents become more aware of their marginalization (Constante et al. 2021), they may need more resources to manage their feelings of distress. In this way, the decline in active coping may reflect broader structural realities that constrain Latinx adolescents' ability to effectively manage stress through problem‐solving. For example, it is plausible that active coping remained stable from 8th to 9th grade because Latinx adolescents had greater access to resources for problem solving that supported their transition and in 10th grade had fewer resources. Therefore, addressing active coping may need to go beyond individual skill development to include efforts that provide resources and foster inclusive environments for Latinx adolescents.

### 6.2 Goal 2: Gender and Nativity Variation

No differences, by gender or nativity, emerged in the trajectory of active coping. As U.S. and foreign‐born Latinx adolescent girls and boys get older they have similar cognitive capacities to understand the outcomes linked to social stratification (e.g., ethnic‐racial discrimination; Constante et al. 2021). It is possible that regardless of gender and nativity, Latinx adolescents may experience a greater frequency of uncontrollable system‐level challenges due to discriminatory policies or limited access to resources (Constante et al. 2021). As there are no immediate solutions to such uncontrollable challenges, active coping which focuses on problem solving may not be effective in alleviating stress. Thus, overtime Latinx adolescents may discover emotion‐focused coping strategies, such as expressing or discussing feelings (Gonzales et al. 2001), is more effective in managing their levels of distress when experiencing uncontrollable challenges. As the findings of the current study suggest active coping declines, it is important to expand these analyses to include a more diverse Latinx sample and perhaps culturally relevant forms of coping (e.g., religious coping).

### 6.3 Limitations and Future Directions

There are some limitations to the current study that should be considered in future research. The current study only used three time points, and the quadratic variance was constrained to zero. Future work should use more than three time points. The inclusion of more time points will allow for a better understanding of within person variability of the quadratic trajectory. Further, the addition of more than three time points can capture the transitions from middle to high school and entrance to emerging adulthood which may identify whether problem solving supports are needed in college settings. Additionally, the study sample size may have been too small and most participants in the current study were born in the United States and live in a specific Southwestern region. Future research should use a larger more nationally representative sample and recruit a somewhat even number of U.S.‐born and foreign‐born Latinx adolescents. A larger and more evenly distributed Latinx sample may allow for better detection of changes in active coping during the transition from 8th to 9th grade and within group differences (e.g., nativity). Moreover, future research should examine differences in the developmental trajectory of active coping by other salient within‐group characteristics such as generational status, and language use. Recognizing within‐group differences is essential for developing prevention and intervention efforts that are responsive to the heterogeneity of needs, experiences, and identities among Latinx adolescents. Despite these limitations the current study provided a new understanding of the ways active coping changed from middle to high school among Latinx adolescents.

### 6.4 Conclusion and Implications

The findings of the current study suggested that the transition from 9th to 10th grade is a sensitive period that relates to decline in active coping. As such, it may be important for intervention programs focused on developing active coping among Latinx adolescents to target the transition from 9th to 10th grade or for prevention programs to target the middle school years. Moreover, as the life course perspective suggests environmental changes may provide a lack of opportunities that engender decline in assets (Alexander et al. 1988; Elder 1998), it can be speculated that the decline in active coping among Latinx adolescents in this study may be due to a lack of school resources. As such, it may be important for prevention and intervention programs focused on fostering active coping to support school level factors such as school policies and availability of resources. The findings also suggest that patterns of change in active coping across the transition from middle to high school were similar for boys and girls and for U.S. born and foreign‐born youth. Therefore, it is important to provide resources that promote the development of active coping strategies (e.g., problem‐solving) for both boys and girls, as well as U.S. born and foreign‐born Latinx adolescents equally during the transition from 9th grade to 10th grade.

## Ethics Statement

The research conducted in this study was reviewed and approved by the Institutional Review Boards at the Texas State University (2014D2548). All aspects of the study were in compliance with APA Ethical Principles.

## Data Availability

The data that support the findings of this study are available from the corresponding author upon reasonable request.
